# Generation and Characterization of Acid Tolerant *Fibrobacter succinogenes* S85

**DOI:** 10.1038/s41598-017-02628-w

**Published:** 2017-05-23

**Authors:** Chia-wei Wu, Thomas Spike, Dawn M. Klingeman, Miguel Rodriguez, Virgil R. Bremer, Steven D. Brown

**Affiliations:** 1 Biosciences Division, Oak Ridge National Laboratory, Oak Ridge, TN USA; 2 Biota Biosciences, Inc., Cambridge City, IN USA; 3 BioEnergy Science Center, Oak Ridge National Laboratory, Oak Ridge, TN USA; 4 Elanco Animal Health, Greenfield, IN USA; 5 Department of Pathobiological Sciences, University of Wisconsin-Madison, Madison, WI USA

## Abstract

Microorganisms are key components for plant biomass breakdown within rumen environments. *Fibrobacter succinogenes* have been identified as being active and dominant cellulolytic members of the rumen. In this study, *F*. *succinogenes* type strain S85 was adapted for steady state growth in continuous culture at pH 5.75 and confirmed to grow in the range of pH 5.60–5.65, which is lower than has been reported previously. Wild type and acid tolerant strains digested corn stover with equal efficiency in batch culture at low pH. RNA-seq analysis revealed 268 and 829 genes were differentially expressed at pH 6.10 and 5.65 compared to pH 6.70, respectively. Resequencing analysis identified seven single nucleotide polymorphisms (SNPs) in the *sufD*, *yidE*, *xylE*, *rlmM*, *mscL* and *dosC* genes of acid tolerant strains. Due to the absence of a *F*. *succinogenes* genetic system, homologues in *Escherichia coli* were mutated and complemented and the resulting strains were assayed for acid survival. Complementation with wild-type or acid tolerant *F*. *succinogenes sufD* restored *E*. *coli* wild-type levels of acid tolerance, suggesting a possible role in acid homeostasis. Recent genetic engineering developments need to be adapted and applied in *F*. *succinogenes* to further our understanding of this bacterium.

## Introduction

Understanding and overcoming plant biomass recalcitrance is a crucial step to enable industrial biofuels and biomaterials production^1, 2^. There is interest in examining and learning from environments where nature’s best lignocellulosic deconstruction occurs. Industrial ethanologenic bacteria and yeast are capable of ethanol production in pH ranges of pH 3.5–7.5 and pH 2–6.5, respectively^3, 4^. In general, anaerobic cellulolytic bacteria are not known to grow (i.e., increase microbial cell mass) below pH 6.0 and the majority of fermentative microorganisms grow within a relatively narrow pH range near circumneutral pH^5^. There is a biotechnological interest in fermentation processes and enzymes that can operate at lower pH values to mitigate contamination and to enhance productivity^6, 7^.

Herbivorous mammals have a variety of specialized compartments to breakdown and to access nutrients derived from dietary plant materials. Efficient biomass breakdown in the rumen is facilitated by a complex consortium of anaerobic microorganisms and host actions^8–10^, and building an expanded gene catalogue for biomass deconstruction is one area of applied interest^11^. Rumen microorganisms produce an abundance of different enzymes necessary for biomass deconstruction, as well as producing short chain (C2 to C6) volatile fatty acids (VFAs) and other compounds that support animal energy requirements^11^. Other microbial metabolic products include lactic acid, methane, hydrogen and carbon dioxide, as well as numerous cross feeding interactions and opportunities among microbiome members.

Several studies have observed large differences in rumen bacterial community composition for animals feeding on the same diet^12, 13^, while other studies indicate steady microbiome core taxa are maintained even as microbial communities shift following diet changes^14, 15^. Modern cattle diets contain rapidly fermentable feedstuffs, which results in low rumen pH for much of the day, reducing the efficiency of forage utilization and can lead to a condition known as sub-acute ruminal acidosis or chronic acidosis^16^. Inefficient and slow anaerobic degradation of cellulose by rumen microbial consortia has been reported as low as pH 5.3 in batch experiments for feedlot-type diets. Major rumen pH decline can reduce populations of cellulolytic bacteria and increase bacterial endotoxins leading to inflammatory responses and a negative impact on animal health^16^. Approaches to attenuate rumen pH decreases have included application of various feed supplements, such as probiotic *Saccharomyces cerevisiae* strain CNCM I-1077^17^, *Megasphaera elsdenii*, strain H6F32^18^, flavonoids^19^, dicarboxylic acids malate and fumarate^20^, and essential oils^21^. Hence, there is interest in better understanding and preventing adverse outcomes associated with sub-acute ruminal acidosis.

In early rumen studies, the Gram positive *Ruminococcus flavefaciens* and *Ruminococcus albus*, and Gram negative *Fibrobacter succinogenes* were identified as being key active cellulolytic members in the rumen^8^. More recently, molecular biology tools have provided new insights into the complex community that resists cultivation and greatly expanded the cow rumen carbohydrate-active gene catalogue^22^. Metagenomic studies have characterized rumen microbial community structure and functions under a variety of conditions^23–25^. Amongst other challenges, obtaining ruminal pH samples and analyses can be complicated as ruminal pH is not homogenous and different analytical techniques can produce different results^26^.

In order to better understand the limitations of a key rumen fermentative microorganism at lower pH, we studied *F*. *succinogenes* type strain S85 as a model organism using chemostat cultivation and systems biology tools. *Fibrobacter* spp. are able to ferment xylan, glucose, cellobiose and cellulose to form succinic, acetic and formic acids^27^. Members of the phylum are found in termite guts and *Fibrobacter* spp. have been detected in diverse terrestrial and aquatic environments. A 3.84 Mb complete genome sequence for *F*. *succinogenes* S85 has been reported^28^. A study with 22 combinations of cellulose-fed dilution rates (D, 0.014–0.076 h^−1^) and pH (6.11–6.84) revealed *F*. *succinogenes* S85 could not maintain steady state growth below pH 6.1 and was washed out of continuous cultivation^29^, consistent with an earlier study where the same strain was washed out of cellobiose grown continuous cultures at pH 6.0 (D, 0.165 h^−1^)^30^. We hypothesized that strain S85 could grow in continuous cultivation below pH 6.0 following strain adaption and might have enhanced properties for biomass deconstruction and fermentation.

## Materials and Methods

### Bacterial strains and culture media

*Fibrobacter succinogenes* S85 (ATCC 19169^T^) was purchased from ATCC although the bacteria used in the experiment were a kind gift from Dr. Garret Suen at University of Wisconsin-Madison. Growth medium for *F*. *succinogenes* S85 was prepared as described previously^28^. Briefly, boiled modified Dehority medium (MDM) was saturated with high purity CO_2_ during cooling to room temperature, anaerobically dispensed and sealed in serum bottles or Hungate tubes, and sterilized by autoclave. Before inoculation, medium amendments were added, including cellobiose at a concentration of 4 g/L unless otherwise specified. The complete medium also contained 0.1% cysteine-HCl as a reducing agent and 0.001% resazurin as an oxygen indicator. Cultures were incubated at 37 °C without shaking.

*Escherichia coli* knockout mutant strains were purchased from the *E*. *coli* Genetic Stock Center (CGSC) at Yale University. The mutant library was originally constructed as the Keio collection^31^. The identity of each mutant strain was confirmed by PCR amplification of the junction region spanning the kanamycin resistance cassette and its upstream region, with the k1 and H1 PCR primers, as described previously^31^.

### Bioreactor setup

*F*. *succinogenes* S85 was grown at 37 °C in a 500-ml (vessel capacity 1.3 L) culture using a water-jacketed BioFlo110 bioreactor (New Brunswick Scientific, Edison, NJ) for continuous growth at different dilution rates over hundreds of hours. Temperature, pH, and agitation were monitored and controlled in the bioreactor via the BioCommand 2.62 software. Continuous culture conditions were similar to those described previously^29, 32^. Briefly, ultrapure N_2_ and CO_2_ gases were connected to a bioreactor gas mixing unit and delivered to the bioreactor’s sparger through a sterile filter and tube. The medium feed line was connected to an anaerobic 19-L glass medium reservoir through a glass 3-way medium break tube. The gas inlet on the medium break tube was slightly pressurized with N_2_ gas to prevent medium from flowing backward. The medium flow rate was controlled with a MINIPULS 3 peristaltic pump (Gilson, Middleton, WI) and was measured by weighing collected effluent over a period of time. Dilution rates were varied throughout the course of the experiment and are described below. The bioreactor and medium feed carboy were kept anaerobic via gas sparging with either N_2_ or CO_2_ gases. Culture pH was maintained and controlled through a combination of gas mixing and acid addition. The controller was set to constantly sparge N_2_ into the culture, which maintained an anaerobic environment and gradually increased the pH due to the buffering property of carbonate in the medium. Once culture pH raised above the set point, the CO_2_ valve was opened by the pH control system and equilibrated the culture pH around the set point within a ±0.01 range. For conditions where pH set points were below carbonate buffering capacity (~pH 6.1), 0.5 N HCl was pumped into the bioreactor by the pH control system. Gel-reference pH electrodes were purchased from Mettler-Toledo (Woburn, MA). After autoclaving of the bioreactor and throughout the course of culturing, the electrodes were regularly checked and calibrated for accuracy, by measuring the pH of a culture sample taken from the bioreactor with a calibrated external pH meter (Fisher Scientific Accumet AB150, Pittsburg, PA). To generate acid tolerant *F*. *succinogenes*, N-methyl-N-nitro-N-nitrosoguanidine (NTG, obtained from TCI America and manufactured by Tokyo Chemical Industry Co. Ltd, Tokyo, Japan)^33–35^ was added to chemostat cultures at 15 µg/ml, and tolerant strains were selected by growth at lower pH based upon previous literature values^29, 30^. The dosage of NTG at 15 µg/ml was chosen based on previous studies for different bacteria, e.g. ref. 36, and also on preliminary experiments where a range of NTG concentrations were tested for its effects on log phase culture in Hungate tubes. Wild-type *F*. *succinogenes* treated at this level had static growth and no significant autolysis measured by optical density readings.

### Biomass pre-treatment, culturing and compositional analysis

Milling of dry corn stover was conducted firstly with a Wiley Mill Model 4 (Thomas Scientific, Swedesboro, NJ) through a 2-mm mesh, and further milled through a number 60 mesh with a Wiley Mini-Mill (Thomas Scientific). The 60-mesh milled corn stover was sieved through a 12-in stainless-steel number 170 mesh (Fisher Scientific, Pittsburgh, PA) on a shaking platform at 300 rpm. The sieved corn stover was added into MDM at 6 g/L in serum bottles, de-gassed, sealed, and autoclaved for 15 min. Before inoculation, amendments, except cellobiose, were added as described above. Inocula were grown in cellobiose batch culture until OD_680_ reached around 1.0, at which time the cultures were washed with oxygen-depleted MDM, resuspended and injected into serum bottles at a density equivalent to OD_680_ 0.1. Carbohydrate content in corn stover was analyzed with a quantitative saccharification assay as described before^37, 38^. Briefly, biomass before or after fermentation was washed to remove bacteria and then hydrolyzed in 72% w/w H_2_SO_4_ for 1 h at 30 °C and was further broken down into oligomers in 4% w/w H_2_SO_4_ at 121 °C for 1 h. Samples were then neutralized to pH ~7.0 with calcium carbonate, filtered through 0.2 µm and carbohydrate content (glucose, xylose, galactose, mannose, and arabinose) was quantified by high-performance liquid chromatography (HPLC) against known standards.

### High-performance liquid chromatography (HPLC) analysis

Concentrations of cellobiose, succinate, formate and acetate in culture samples were analyzed with HPLC. Samples taken at different time points were stored at −20 °C. Before HPLC analysis, the samples were thawed, filtered through a 0.2-μm nylon filter and acidified with 2 M sulfuric acid. Twenty microliters each of the acidified samples were then applied to an Aminex HPX-87H, 300 × 7.8 mm column (Bio-Rad, Hercules, CA) on a Hitachi LaChrom Elite System (Hitachi High Technologies America, Inc., San Jose, CA) or a Waters Breeze system (Waters Corp., Milford, MA). Analysis was performed at a flow rate of 0.5 ml/min in 5 mM H_2_SO_4_ for 35 min as previously described^39^. Soluble fermentation products were identified by comparison with retention times and peak areas of corresponding standards.

### Genomic DNA isolation and preparation for resequencing or PCR

Cells were pelleted from a 50 ml chemostat sample by centrifugation (7,600 × *g*, 4 °C for 4 min), flash frozen in liquid nitrogen, and stored at −80 °C. The genomic DNA (gDNA) extraction protocol was adapted from Current Protocols in Molecular Biology^40^. Briefly, a solution of 565 µl of TE buffer, 2 µl of 100 mg/ml RNase A, 30 µl of 10% SDS and 3 µl of 20 mg/ml proteinase K was added to a bacterial pellet and the well-mixed suspension was incubated at 65 °C for 1 h. A 100 µl aliquot of 5 M NaCl and 80 µl of 4.2% NaCl/10% cetyltrimethylammonium bromide (CTAB) were sequentially added and mixed into the bacterial lysate, followed by a 10-min incubation at 65 °C and two rounds of phenol/chloroform extractions. Genomic DNA was precipitated with 0.7 × volume of isopropanol, washed with 70% ethanol, dried in a DNA mini speed vacuum (ATR, Laurel, MD), and finally resuspended in deionized H_2_O. DNA quality was assessed by NanoDrop (Thermo Fisher Scientific Inc.) and quantity was determined by the Qubit broad range double stranded DNA assay (Life Technologies, Grand Island, NY). Illumina TruSeq libraries for resequencing were prepared from gDNA as described in the manufacturer’s protocols following the low throughput protocol. Final libraries were validated by Qubit and visualized with Agilent 2100 Bioanalyzer (Agilent Technologies Santa Clara, CA) for appearance and size determination. Samples were normalized using the Illumina’s Library dilution calculator and pooled into a 10 nM stock.

### Illumina sequence generation and analysis

Samples were sequenced on an Ilumina MiSeq Instrument (Illumina, San Diego, CA). Paired end sequencing (2 × 251) was completed on the TruSeq genomic DNA libraries with a MiSeq V2 cartridge. Sequencing reads raw data were imported to CLC Genomics Workbench 6.5 (CLC bio, Boston, MA), trimmed, and mapped to *F*. *succinogenes* S85 genome sequence (CP001792). The trim settings for the raw data inputs used for removal of low quality sequences were: limit = 0.02, removal of ambiguous nucleotides: maximal 1 nucleotide allowed, and removal of sequences on length: minimal 20 nucleotides. Genome sequence variations were detected with the quality-based variation detection tool, requiring presence in both forward and reverse reads and at least 10 times reads coverage. Minimum neighborhood and central quality were set at 15 and 20, respectively.

### RNA extraction, RNA-seq library construction and analysis

Cells were pelleted from a 50 ml chemostat sample by centrifugation (7,600 × *g*, 4 °C for 4 min), flash frozen in liquid nitrogen, and stored at −80 °C. Ten milliliters of TRIzol reagent (Life Technologies) were added to the frozen cell pellet and mixed by vortexing until the pellet was fully dissolved. After treating with chloroform, the aqueous phase containing RNA was further processed with a Qiagen RNeasy mini kit (Qiagen, Valencia, CA), following the manufacturer’s instructions, including the on-column DNase I (Qiagen) treatment. Quantity of RNA was measured with a NanoDrop spectrophotometer and quality was assessed with an Agilent Bioanalyzer (Agilent Technologies, Santa Clara, CA) on a RNA 6000 Nano Chip. Ribosomal RNA was depleted using the Ribo-Zero rRNA Removal Kit for Gram-negative Bacteria (Epicentre, Madison, WI) following the manufacturer’s protocol. RNA-seq libraries were constructed using an Epicentre Scriptseq mRNA-Seq library preparation kit (Illumina compatible). Briefly, as previously described (1), depleted RNA samples were used to synthesize barcode-tagged cDNA. The final RNA-seq libraries were quantified with a Qubit Fluorometer and library quality was assessed with Agilent Bioanalyzer. Final libraries were normalized using the Illumina’s library dilution calculator and pooled into a 10 nM stock. Trimmed raw count data for uniquely mapping reads underwent negative binomial distribution normalization and statistical analysis in the DESeq2 software^41^. Differential expression relative to the reference condition, pH 6.70, was defined as genes with a fold of change ≥2 for up-regulation, or ≤−2 for down-regulation, and with an adjusted *P*-value ≤ 0.05 based on DESeq2 analysis.

A gene ontology enrichment analysis was performed on differentially expressed genes using the 2014 version of Clusters of Orthologous Group (COG) database^42^ (available at ftp://ftp.ncbi.nih.gov/pub/COG/COG2014/data). Genes that showed differential expression were grouped into their COG categories. To examine which COG categories were over-represented in differentially expressed genes, a Fisher’s exact test was used. The significance level α was adjusted by Bonferroni correction.

### Plasmid construction

Expression vector pBAD24 was used in the current study. It is inducible with a wide range of arabinose concentrations and expression levels are extremely low in the absence of arabinose^43^. Purified plasmid DNA were digested with *Xba*I and *Sda*I within the multiple cloning site, treated with calf intestinal alkaline phosphatase (Promega) and recovered after agarose gel electrophoresis with Zymoclean Gel DNA Recovery Kit (Zymo Research, Irvine, CA). Insert fragments were amplified with PCR reactions from gDNA of *E*. *coli* BW25113, wild-type *F*. *succinogenes* S85 or acid-tolerant *F*. *succinogenes*. Primers used for PCR amplifications were listed as follows: *E*. *coli sigS* sequence, Fwd, 5′-ACTCTAGAGAGTCAGAATACGCTGAAAGTTCA, Rev, 5′-ACCCTGCAGGTGAGACTGGCCTTTCTGACA; *E*. *coli sufD*, Fwd, 5′-ACTCTAGAGGCTGGCTTACCGAACAGC, Rev, 5′-ACCCTGCAGGCCCGCACTTTGTCG; *E*. *coli sufDSE*, Fwd, 5′-ACTCTAGAGGCTGGCTTACCGAACAGC, Rev, 5′-ACCCTGCAGGAGCCAACCGGATGAAAGC. The thermocycler program was set for an initial cycle at 94 °C for 3 min, 25 cycles of 94 °C for 30 sec, 56 °C for 30 sec and 72 °C for 60–150 sec, depending on insert size. DNA polymerase for the reactions was Platinum® *Taq* DNA Polymerase High Fidelity (Life Technologies, Carlsbad, CA). Amplified insert fragments were purified with DNA Clean & Concentrator columns (Zymo Research), digested with *Xba*I and *Sda*I and purified again with DNA Clean & Concentrator columns. The purified inserts were ligated (T4 DNA ligase, New England BioLabs, Ipswich, MA) into the processed pBAD24, then the ligation mixture was transformed by heat shock into appropriate host competent cells. For constructs that contain coding sequences from both *F*. *succinogenes* and *E*. *coli*, the plasmid DNA of pBAD24-*sufDSE*, constructed as described above, was digested with *Xba*I and *Sal*I to remove the whole *sufD* coding sequence and the first 13 bp of the downstream gene, *sufS*. An insert fragment amplified from either the wild-type or acid-tolerant *F*. *succinogenes* gDNA with primer pair Fwd, 5′-ACTCTAGAGAACGCTGAATTTATACAGAACTTGC and Rev, 5′-ACGTCGACGGAAAAAATCATCTTGCACCTCCTGGAATTTCCGCCATCAATCGC was digested with *Xba*I and *Sal*I, purified and ligated into the processed pBAD24-*sufSE* to create a fusion operon containing *F*. *succinogenes sufD* and *E*. *coli sufSE* genes. The reverse primer for these constructs included the 3′ end sequences of *F*. *succinogenes sufD*, the endogenous Shine-Dalgarno sequence and the first 13 bp of *sufS*. As a result, this fusion operon had full-length *F*. *succinogenes sufD* fused with the last 18 bp of *E*. *coli sufD*, which contained the Shine-Dalgarno sequence, and in-frame *E*. *coli sufS* and *sufE*.

## Results

### Culturing and generating acid tolerant *F*. *succinogenes* strains

To culture *F*. *succinogenes* at a 500-ml scale in a bioreactor, wild-type *F*. *succinogenes* S85 was first grown overnight in a serum bottle with 50 ml of medium supplemented with 3 g/L cellobiose, which was then inoculated into a bioreactor that contained 500 ml of fully reduced complete medium at pH 6.70. The bioreactor was initially run in batch mode and until the culture reached early stationary phase (OD_680_ around 1.3), around which point chemostat growth was initiated by continuous input of medium containing 3 g/L cellobiose, at 970 ml/day (D = 0.08 h^−1^). Initially, the chemostat was set at pH 6.70 and maintained using dynamic mixing of N_2_ and CO_2_ gases. Under such conditions the culture maintained a stable cell density with OD_680_ readings around 1.3 for at least 5 volume changes and was considered growing in steady state. To monitor cellobiose consumption and succinate accumulation in the bioreactor, samples were analyzed by HPLC. Succinate concentration during steady state was around 11.5 mM. The pH set point was then lowered to 6.25.

**Figure 1:**
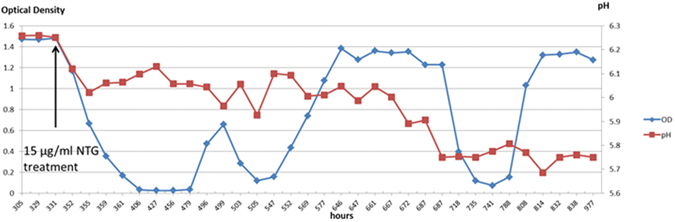
*F*. *succinogenes* growth and pH control after random mutagenesis. When the chemostat culture of wild-type *F*. *succinogenes* was grown in steady state in cellobiose medium at pH 6.25, mutagen NTG was added to induce random mutations. The pH and medium feed control units were carefully controlled to maintain viability of the culture and to screen for acid tolerant populations. As pH (red) decreased over time, the OD readings in blue shows culture adaptation to low pH stress accordingly. The mutant populations went through three major adjustments and finally reached steady state at pH 5.75. The selected culture was later confirmed to have the ability to tolerate a pH between 5.60 and 5.65 in batch culture.

The pH 6.25 steady-state culture was treated with NTG at 15 µg/ml to begin the selection of acid tolerant strains^33–35^ (Fig. 1). At the time NTG was added into the bioreactor, medium feed was stopped for the following hour, and then restarted at half the dilution rate (D = 0.04 h^−1^) as prior to the treatment. To create an acidic environment and select for acid tolerant strains, the chemostat culture was adjusted with HCl to pH 6.05 at 21 h post-NTG treatment, near the pH value (pH 6.1) that is thought to be the lower limit of ruminal bacteria growth in continuous culturing^29, 30, 44^. Cell density was observed to decline steadily from OD_680_ 1.48 at the time of treatment to OD_680_ 0.27 at 30 h post-treatment, which suggested cells had undergone static growth and were being washed out, and some proportion of cells also likely underwent lysis. The medium feed was then stopped, and 250 ml of fresh medium was added to dilute down any possible detrimental metabolites or residual NTG. A continued decline in culture OD_680_ readings was observed until below OD_680_ 0.05 at 53 h post-treatment, while at pH 6.05. Significant growth resumed at 148 h post-treatment with the culture density reaching OD_680_ 0.66, at which time the medium feed was reestablished at a dilution rate of around 0.04 h^−1^. Another drop in cell density occurred around 169 h post-treatment (Fig. 1) and growth recovered within 40 hours by using the same approaches described above. After culture recovery, culture pH was gradually lowered below 6.0 in steps of pH values of 0.05 or 0.10 over a 30-hour period. The culture density was maintained around OD_680_ 1.3 until the pH was lowered to 5.75, when it dropped below OD_680_ 0.05 and soon recovered again (Fig. 1) remaining stable at OD_680_ ~ 1.3 (Fig. 1).

**Figure 2:**
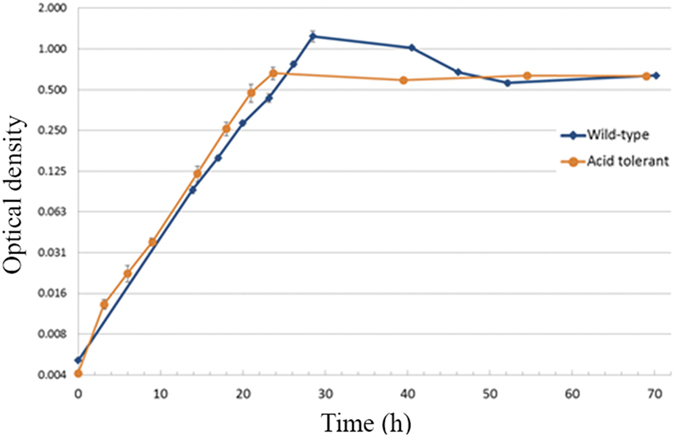
Growth curves of wild-type and acid tolerant strains of *F*. *succinogenes* in cellobiose medium. A growth curve of the acid tolerant *F*. *succinogenes* grown in 3 g/L cellobiose medium (initial pH 6.90 was measured by cell density readings and plotted against the wild type. The two had the same doubling time but the wild type reached a higher density (OD > 1.2) in late log phase while the acid-tolerant culture grew to OD around 0.7.

**Figure 3:**
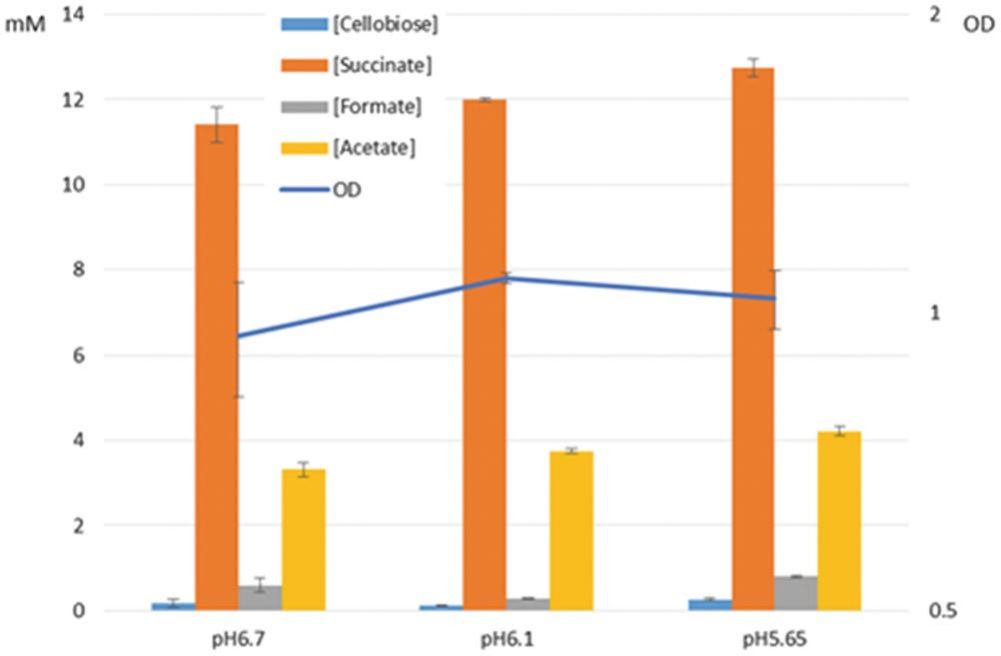
Metabolic profiles of chemostat culture in steady state at different pH values. After the chemostat culture reached steady state at the indicated pH, samples were taken for HPLC analysis. At least three samples per pH setting were analyzed. Concentrations of succinate and acetate in the culture increased as culture pH decreased, while pH 6.10 samples had the least formate concentration among the three. Concentration of cellobiose was also the highest at pH 5.65, suggesting a slight decrease of cellobiose intake or utilization despite the relatively constant cell density across conditions.

Overall, after NTG treatment, the culture went through three major growth fluctuations along a series of adjustments of decreasing pH set points during a course of ~500 h. After the stable acid tolerant population had been obtained, aliquots were harvested, stored at −80 °C and were later able to be recovered in batch culture in medium pre-adjusted at pH 6.70 and pH 5.70, while the wild-type strain could only grow in pH 6.70 medium. In batch culture at pH 6.70, wild-type *F*. *succinogenes* S85 appeared to have a higher stationary phase cell density (OD_680_ around 1.2), while the acid tolerant strain had density readings around 0.68. Nevertheless, the doubling times for both strains were not different (Fig. 2). The selected *F*. *succinogenes* acid tolerant mutant population was later grown in a chemostat and tested for their pH lower limit. In the same chemostat settings (3 g/L cellobiose, D = 0.08 h^−1^), the culture successfully reached steady state at pH 5.65 and cell density readings remained stable around 1.0 for 148 h until the setpoint was further lowered to pH 5.60. The cell density then dropped below OD_680_ 0.1 and thus the culture was unable to tolerate the new levels of acidity. Therefore, the acid tolerant *F*. *succinogenes* had a pH lower limit between pH 5.60 and pH 5.65. Metabolite profiles for cellobiose, succinate, formate and acetate in steady state at three pH set points were analyzed with HPLC (Fig. 3). The cellobiose concentration in samples from the pH 5.65 chemostat was higher (0.27 mM) than those at pH 6.70 and pH 6.10 (0.17 and 0.13 mM, respectively). Succinate concentrations increased from 11.4 mM, 12.0 mM to 12.7 mM as pH decreased from 6.70 to 6.10, and 5.65, respectively. Acetate had similar increases to succinate while formate dropped from 0.6 mM to 0.29 mM and rose to 0.8 mM at pH 6.70, 6.10 and 5.65, respectively.

### Biomass fermentation of the acid tolerant *F*. *succinogenes* strains

We compared the ability of *F*. *succinogenes* to digest fiber between wild type and acid tolerant strains in batch culture containing corn stover as the carbon source. Batch culture succinate concentrations in medium were measured as an indication of bacterial growth. In medium at pH 6.70, succinate concentration up to 72 h post-inoculation did not significantly differ between wild type and acid tolerant strains (*P* = 0.4). Similarly, wild type and acid tolerant strains inoculated into medium at pH 5.75 produced comparable amount of succinate (*P* = 0.4). However, the succinate concentration at 72 h in the high pH environment was about 2.8 times higher than that in the low pH regardless of the strains inoculated. We further examined sugar composition of the biomass before and after fermentation with a quantitative saccharification assay, a method to hydrolyze polysaccharides and analyze oligomer components from the samples. This analysis showed that there was no significant difference of glucose, xylose and mannose content in the remaining biomass after fermentation (7 days post inoculation) among samples from wild type and acid tolerant strains regardless of the pH of the medium (Supplementary Fig. 1). On the other hand, there was less galactose in the remaining biomass at pH 6.70 than at pH 5.75 when compared between the same strain (*P* = 0.008 between wild type and 0.02 between acid tolerant strain). Similarly, arabinose content was found to be less in samples at pH 6.70 than pH 5.75 (*P* = 3.6E-05 between wild type and 7.6E-04 between acid tolerant strain). However, there was no significant difference of sugar contents in corn stover after fermentation between wild type and acid tolerant strains at pH 5.75 (Supplementary Fig. 1), suggesting the acid tolerant strains digested corn stover in a similar manner to the wild type at low pH environments.

### Genome sequence analysis

To identify variations in the acid tolerant strains, gDNA was prepared from a chemostat culture sample taken at pH 5.65 (time mark EFT723). Genomic DNA from wild-type *F*. *succinogenes* S85 was also prepared in parallel. This wild-type culture had been grown for two generations after we acquired it. Resequencing of the wild-type and acid tolerant populations generated overall genome sequence coverages of 398× and 309×, respectively. Raw data are available via the NCBI Sequence Read Archive (SRA) under accession SRP097623. A quality-based variant analysis was used to identify sequence variations in the wild-type and acid-tolerant strains using the published *F*. *succinogenes* S85 (CP001792) reference sequence. Table 1 lists all the detected variations (N = 8) between our wild-type strain sequence and the reference strain sequence, six of which are within coding regions. Two of the six variations are consecutive substitutions within the coding region of Fisuc_0822, an FG-GAP repeat protein gene^45, 46^ based on sequence homology. This results in a synonymous substitution at residue 1223 and a valine-to-isoleucine substitution at residue 1224. Another sequence variation was found within the coding region of Fisuc_1012, a gene encoding a branched-chain amino acid transport protein, AzlC, where the threonine at the 48^th^ residue is substituted with an alanine. The remaining three variations within the coding regions of Fisuc_2022 and Fisuc_3045 are synonymous (Table 1). In addition, two single nucleotide insertions were identified in intergenic regions between Fisuc_2307 and Fisuc_2308, as well as between Fisuc_2832 and Fisuc_2833. The former insertion is an A, 45 bp and 141 bp away from the initiation codons of Fisuc_2307 and Fisuc_2308, respectively,as Fisuc_2307 is on the complement strand and Fisuc_2308 is on the forward strand. The other insertion is a T, located in the downstream region of both genes, 151 bp and 117 bp away from the stop codons of Fisuc_2832 and Fisuc_2833, respectively.

**Table 1:** Variations in sequences between published *F*. *succinogenes* S85 and re-sequencing of *F*. *succinogenes* S85 in this study.

Number	Coordinate on CP001792	Locus Tag	Annotation	ORF direction	Position at ORF sequence	Sequence on CP001792	Sequence determined in this study	Count	Coverage	Frequency	Average quality	Distance to initiation codon	residue change
**Coding region**													
1	1007759	Fisuc_0822	FG-GAP repeat protein	—	3670	C	T	115	116	99.1	36.87		Val1224Ile
2	1007760	Fisuc_0822	FG-GAP repeat protein	—	3669	C	G	115	116	99.1	36.87		Thr1223, synonymous
3	1249999	Fisuc_1012	AzlC family protein	—	142	T	C	179	237	75.5	37.483		Thr48Ala
4	2498380	Fisuc_2022	Hypothetical	+	2847	C	T	102	282	36.2	38.126		Asp949, synonymous
5	2498437	Fisuc_2022	Hypothetical	+	2904	T	G	123	265	46.4	35.132		Thr968, synonymous
6	3746851	Fisuc_3045	Tryptophan synthase subunit	+	1029	G	T	155	207	74.9	36.766		Ala343, synonymous
**Noncoding region**													
1	2842868	Intergenic (Fisuc_2307-Fisuc_2308)				—	A	200	217	92.2	37.715	45, 141	
2	3495925	Intergenic (Fisuc_2832, Fisuc_2833)				—	T	144	160	90.0	37.521	N/A	

Seven single nucleotide variations were identified in the acid tolerant culture compared to the published *F*. *succinogenes* S85 sequence (Table 2). One variation is a deletion of G at nucleotide position 462 in the Fisuc_1133 coding sequence (a putative *yidE* permease gene). This deletion results in a frameshift mutation, generating a stop codon at the 18th codon position after the deletion. The mutant gene therefore has only 172 codons instead of 555 codons. The remaining six variations are single nucleotide substitutions, resulting in six residue changes in five genes, Fisuc_0527, Fisuc_1804, Fisuc_1945, Fisuc_2074 and Fisuc_2957, encoding SufD, XylE, RlmM, MscL and DosC, respectively.

**Table 2:** Sequence variations between the acid tolerant strain and wild type of *F*. *succinogenes* S85.

Number	Coordinate on CP001792	Locus Tag	Gene name	Annotation	ORF direction	Position at ORF sequence	Sequence on CP001792	Sequence determined in this study	Count	Coverage	Frequency	residue change	Gene homolog in *E*. *coli*
1	612124	Fisuc_0527	*sufD*	ABC-type transport system involved in Fe-S cluster assembly, permease component	+	514	C	T	61	151	63.04	Arg172Cys	JW1671 (b1681)
2	1396377	Fisuc_1133	*yidE*	Predicted permease, may bind an unidentified ligand	−	462	G	−	200	200	100	Frameshift. Stop codon at the 18th codon after the shift.	JW3662 (b3685)
3	2230314	Fisuc_1804	*xylE*	sugar transporter	−	205	C	T	217	220	96.14	Ala69Thr	JW3991 (b4031)
4	2399135	Fisuc_1945	*rlmM* (*ygdE*)	DNA alkylation repair	+	331	A	G	199	199	94.12	Thr111Ala	JW2777 (b2806)
5	2561445	Fisuc_2074	*mscL*	Large conductance mechanosensitive channel protein. Critical roles in transducing physical stresses at cell membrane into an electrochemical response	−	310	T	C	156	182	93	Met104Val	JW3252 (b3291)
6	2561669	Fisuc_2074	*mscL*	See above	−	86	C	A	206	206	100	Gly29Val	JW3252 (b3291)
7	3649525	Fisuc_2957	*dosC*	Signal transducer; phosphorylation receiver or oxygen sensing	+	803	G	T	230	232	100	Gly268Val	JW5241 (b1490)

### RNA-seq analysis

**Figure 4:**
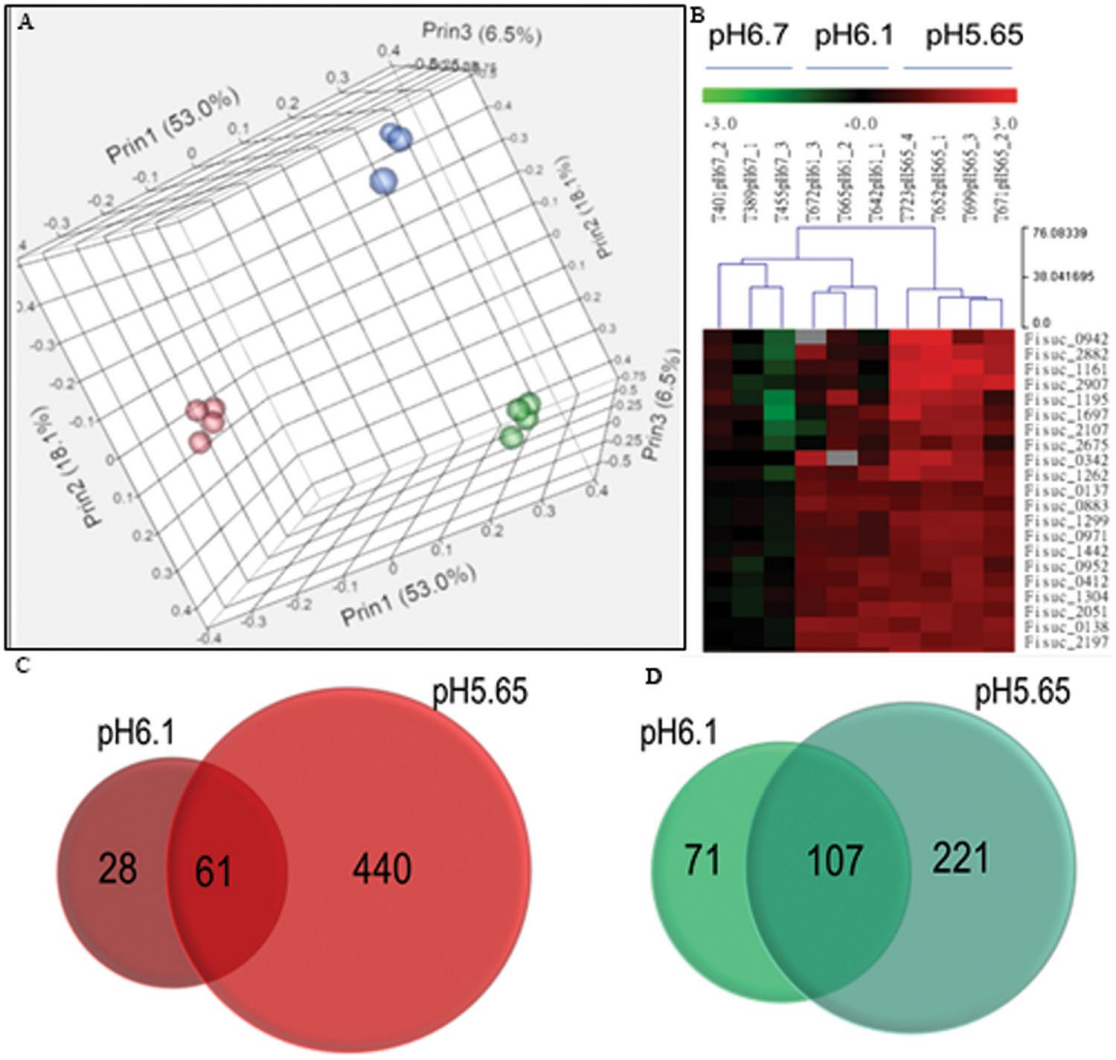
Overview of RNA-seq analysis of chemostat culture under low pH environment. (**A**) A principal component analysis (PCA) plot of the RNA-seq samples at three different pH levels. The close spatial arrangements of samples within each condition indicate high correlation among replicates and the distances among conditions suggest distinct gene expression profiles among conditions. (**B**) A partial hierarchical clustering analysis heat map of all 10 samples. Gene clustering was determined by expression level of each gene but the detailed tree structure was omitted in this graph due to complexity. (**C**) Numbers of genes that were significantly up-regulated (red) or (**D**) down-regulated (green) in pH 6.10 and pH 5.65. Numbers in the overlapped area between two circles indicate numbers of genes that were commonly regulated in both pH.

To gain further insights into *F*. *succinogenes* physiology and gene regulation in acidic environments, global transcript profiles were generated with RNA-seq from chemostat steady state cultures at various pH set points. Transcript profiles of three samples each at pH 6.70 and 6.10 from wild-type *F*. *succinogenes* S85 chemostat were analyzed, as well as for four samples at pH 5.65 from the acid tolerant *F*. *succinogenes* S85 chemostat. RNA-seq data have been deposited in NCBI Gene Expression Omnibus (GEO) database under accession number GSE93907 and raw sequence data deposited at the NCBI SRA under accession number SRP097575. The number of reads, percentage trimmed and uniquely mapped reads, as well as normalized data are provided (Supplementary Dataset Number 1). The range of reads numbers were from 1,763,076 to 4,524,604 among all samples. Trimmed raw count data for uniquely mapping reads underwent negative binomial distribution normalization and statistical analyses in the DESeq2 software^41^. The overall correlation among biological replicates within each pH value and across all three pH conditions is shown in a principal component analysis (PCA) plot (Fig. 4A). The replicates for each condition clustered close to one another for the most part. The first principal component (55.5%) indicates that the mutant S85 chemostat culture at pH 5.65 was the largest variance factor. A hierarchical clustering analysis of the ten samples also demonstrates that samples within each condition grouped closely (Fig. 4B).

Differential expression relative to the reference condition at pH 6.70 identified 89 and 501 genes as being upregulated in pH 6.10 and 5.65 conditions, respectively, among which 61 are common between the two conditions (Fig. 4C). On the other hand, 178 and 328 genes were down-regulated, respectively in pH 6.10 and 5.65 conditions, with 107 common genes (Fig. 4D). All differential expression results are provided (Supplementary Dataset Number 1). The common up-regulated genes between pH 6.10 and 5.65 conditions include 31 hypothetical genes, a heat shock protein Hsp20 (Fisuc_1188), putative transcription regulators Fisuc_0335, Fisuc_0933 and Fisuc_1186, a diguanylate cyclase Fisuc_2957, and genes Fisuc_0137, Fisuc_0138 and Fisuc_2558, which are involved in tryptophan metabolism.

A gene ontology enrichment analysis was performed on differentially expressed genes. Among the up-regulated genes in the pH 5.65 samples, category V: defense mechanisms and category X: Mobilome: prophages, transposons were over-represented (P = 3.27E-06 and 8.00E-04, respectively) (Table 3). Two COG categories (G: Carbohydrate transport and metabolism and Q: Secondary metabolites biosynthesis, transport, and catabolism) were significantly down-regulated in the pH 5.65 samples. Four categories were over-represented from samples collected at pH 5.65 (C: Energy production and conversion, E: Amino acid transport and metabolism, G: Carbohydrate transport and metabolism, and T: Signal transduction mechanisms; Table 4). A list of all 162*F*. *succinogenes* genes annotated in category G was loaded into the KEGG database and 46 genes in the list were returned in the KEGG pathway analysis results. Among these 46 genes, the most up-regulated genes were Fisuc_1985 (1.4 fold at pH 5.65) and Fisuc_1572 (1.3 fold at pH 5.65). Fisuc_1985 is an annotated glycosidase that is involved in glycosaminoglycan degradation and beta-Lactam resistance. Conversely, an orthologous gene (Fisuc_1751) in the same category that was also annotated as K01207, was otherwise found down-regulated (1.8 fold) at pH 5.65. On the other hand, the majority of KEGG-annotated category G genes were down-regulated. Several genes down-regulated by more than 2 fold at pH 5.65 were found involved in glycolysis, such as Fisuc_1204 (glucose-6-phosphate isomerase), Fisuc_2891 (fructose-bisphosphate aldolase), Fisuc_0381 (phosphoglycerate kinase) and Fisuc_1261 (enolase). In the same carbon metabolism KEGG pathway: fsu01200, other genes that were down-regulated between 1.5 and 2 fold included Fisuc_1107 (glucokinase), Fisuc_1322 (6-phosphofructokinase 1), Fisuc_0400 (transketolase), Fisuc_0768 (2,3-bisphosphoglycerate-independent phosphoglycerate mutase) and Fisuc_0024 (ribose 5-phosphate isomerase A). These results suggest that *F*. *succinogenes* acid tolerant strains reduce the activity of the glycolysis pathway for producing pyruvate at low pH environments. We did not observe a known alternative pathway that was activated to compensate the energy production reduction through glycolysis.

**Table 3:** Enrichment analysis of up-regulated genes in low pH environment.

	Category	Number of genes	p-value^a^
pH 5.65	V, Defense mechanisms	24 out of 79	3.27E-06
	X, Mobilome: prophages, transposons	5 out of 8	8.00E-04

^a^Two-tail fisher’s Exact test.

**Table 4:** Enrichment analysis of down-regulated genes in low pH environment.

	Category	Number of genes	p-value^a^
pH 6.10	G, Carbohydrate transport and metabolism	26 out of 162	6.80E-07
	Q, Secondary metabolites biosynthesis, transport and catabolism	8 out of 34	4.99E-04
	C, Energy production and conversion	22 out of 100	6.26E-04
pH 5.65	E, Amino acid transport and metabolism	35 out of 188	4.63E-04
	G, Carbohydrate transport and metabolism	45 out of 162	1.28E-10
	T, Signal transduction mechanisms	4 out of 165	1.02E-04

Other genes possibly involved in carbon metabolism include a group of cellulases that were identified earlier^28^. Expression levels of those 31 cellulases in low pH conditions were compared to those at pH 6.70. At pH 5.65, only two cellulases, Fisuc_1661 (GH5 family endoglucanase^28^) and Fisuc_1426 (GH45 family xylanase^47^), were expressed differently relative to pH 6.70 (2.2 and 1.9 fold, respectively). Eleven cellulase genes were expressed within ±1.5 fold compared to pH 6.70. The other 18 cellulase genes were down-regulated more than 1.5 fold. The expression profiles for these genes at pH 6.10 were similar to pH 5.65, with only one gene up-regulated close to 2 fold (Fisuc_2033, 1.7 fold) and 13 genes down-regulated more than 1.5 fold (Supplementary Dataset Number 1). Before *F*. *succinogenes* digests cellulose, it is thought that it needs to adhere to the surface of cellulose^28, 48, 49^. A group of 10 proteins (Fisuc_0377, Fisuc_1474, Fisuc_1475, Fisuc_1326, Fisuc_1327, Fisuc_1979, Fisuc_2031, Fisuc_2293, Fisuc_2471 and Fisuc_2484) were identified and annotated to have fibro-slime domains and thought to be involved in adherence^28, 50^. In our analysis, only Fisuc_2471 was up-regulated by almost 2 fold at pH 6.10, five genes were moderately up-regulated (1.2–1.4 fold) and four genes were down-regulated (1.02–4.56 fold). At pH 5.65, Fisuc_2471 was the only up-regulated gene (1.4 fold) and the remaining nine genes were all down-regulated (1.1–9.5 fold).

A group of highly differentially expressed genes that drew our attention were genes encoding proteins with Fe-S clusters, specifically radical SAM family proteins. One of the mutations we identified in the acid tolerant *F*. *succinogenes* genome sequence is in the *sufD* gene, encoding a structural protein that may be involved in transfer of sulfur groups to form Fe-S clusters in the SufBCD complex^51, 52^. Fe-S cluster-containing proteins are involved in a pleiotropic range of cellular functions, such as gene regulation, metabolism, and electron transfer. Radical SAM proteins may be one of the groups of proteins under control of Suf and they perform functions such as methylation, isomerization, sulfur insertion, ring formation, anaerobic oxidation and protein radical formation^53^. Of the six significantly expressed radical SAM proteins at pH 5.65, four were up-regulated between 2.0 and 2.4 fold (Fisuc_1354, Fisuc_2052, Fisuc_2935 and Fisuc_2951) and the other two, Fisuc_0219 and Fisuc_2711, were down-regulated (2.8 and 2.4 fold). At pH 6.10, only one of the six genes was significantly up-regulated (Fisuc_2052, 2.1 fold). Analysis of differential gene results for cells collected pH 5.65 compared to pH 6.97 indicate a range of metabolic systems are involved in responding and adapting to growth in lower pH environments (Table 5). Additional RNA-seq analysis is provided as Supplementary Information.

**Table 5:** Selected genes with significantly higher expression levels in pH 5.65 compared to pH 6.70.

Locus Tag	log2 Fold Change	Adjusted P-value	Product Description (with TIGR or COG descriptions)
Fisuc_0029	1.3	2.94E-09	lipoprotein (TIGR02167 bacterial surface protein 26-residue repeat)
Fisuc_0051	2.1	7.37E-13	amidophosphoribosyltransferase (Glutamine phosphoribosylpyrophosphate amidotransferase)
Fisuc_0060	1.4	2.34E-05	chaperonin Cpn10
Fisuc_0159	3.2	2.93E-28	Biotin synthase
Fisuc_0160	3.3	8.63E-13	hypothetical protein (Putative threonine efflux protein)
Fisuc_0163	2.4	3.21E-07	SirA family protein (Predicted redox protein, regulator of disulfide bond formation)
Fisuc_0539	2.4	0.000305	type II and III secretion system protein (component PulD)
Fisuc_0582	2.1	2.47E-10	Sporulation protein YtfJ
Fisuc_1002	2.8	7.52E-16	Phosphotransferase system, phosphocarrier protein HPr
Fisuc_1095	1.8	0.02141	Pyridoxal-5′-phosphate-dependent protein beta subunit (Cysteine synthase)
Fisuc_1133	1.4	8.92E-08	YidE/YbjL duplication
Fisuc_1215	1.6	0.0242	small multidrug resistance protein (Membrane transporters of cations and cationic drugs)
Fisuc_1277	3.1	7.56E-36	preprotein translocase, SecE subunit
Fisuc_1362	1.4	1.18E-05	lipoprotein (TIGR02167 bacterial surface protein 26-residue repeat)
Fisuc_1369	1.9	4.00E-11	Rubredoxin-type Fe(Cys)4 protein
Fisuc_1491	2.4	1.41E-12	NADH-ubiquinone/plastoquinone oxidoreductase chain 3
Fisuc_1532	2.1	5.22E-09	Chorismate binding-like protein (Anthranilate/para-aminobenzoate synthases component I)
Fisuc_1564	2.8	4.71E-07	hypothetical protein (Predicted Na+-dependent transporter)
Fisuc_1593	2.6	1.4E-07	GtrA family protein (Predicted membrane protein)
Fisuc_1665	5.1	1.70E-96	phosphoadenosine phosphosulfate reductase
Fisuc_1668	4.6	1.53E-40	cysteine desulfurase family protein
Fisuc_1673	3.7	4.34E-52	putative RNA polymerase, sigma 70 family subunit
Fisuc_1758	1.3	1.57E-14	hypothetical protein (Cell envelope:Other)
Fisuc_1818	1.9	0.003094	glutamate synthase, NADH/NADPH, small subunit
Fisuc_1820	1.5	0.02261	ammonium transporter
Fisuc_1821	3.0	1.36E-09	nitrogen regulatory protein P-II
Fisuc_2091	2.5	0.001303	Rubredoxin-type Fe(Cys)4 protein
Fisuc_2123	2.3	0.002766	4Fe-4S ferredoxin iron-sulfur binding domain protein
Fisuc_2558	1.9	2.99E-15	Chorismate mutase
Fisuc_2559	2.0	1.03E-16	Prephenate dehydrogenase
Fisuc_2908	2.1	0.000191	(Sulfur transfer protein involved in) thiamine biosynthesis protein ThiS

### Acid survival for *E*. *coli* knockout mutants

**Figure 5:**
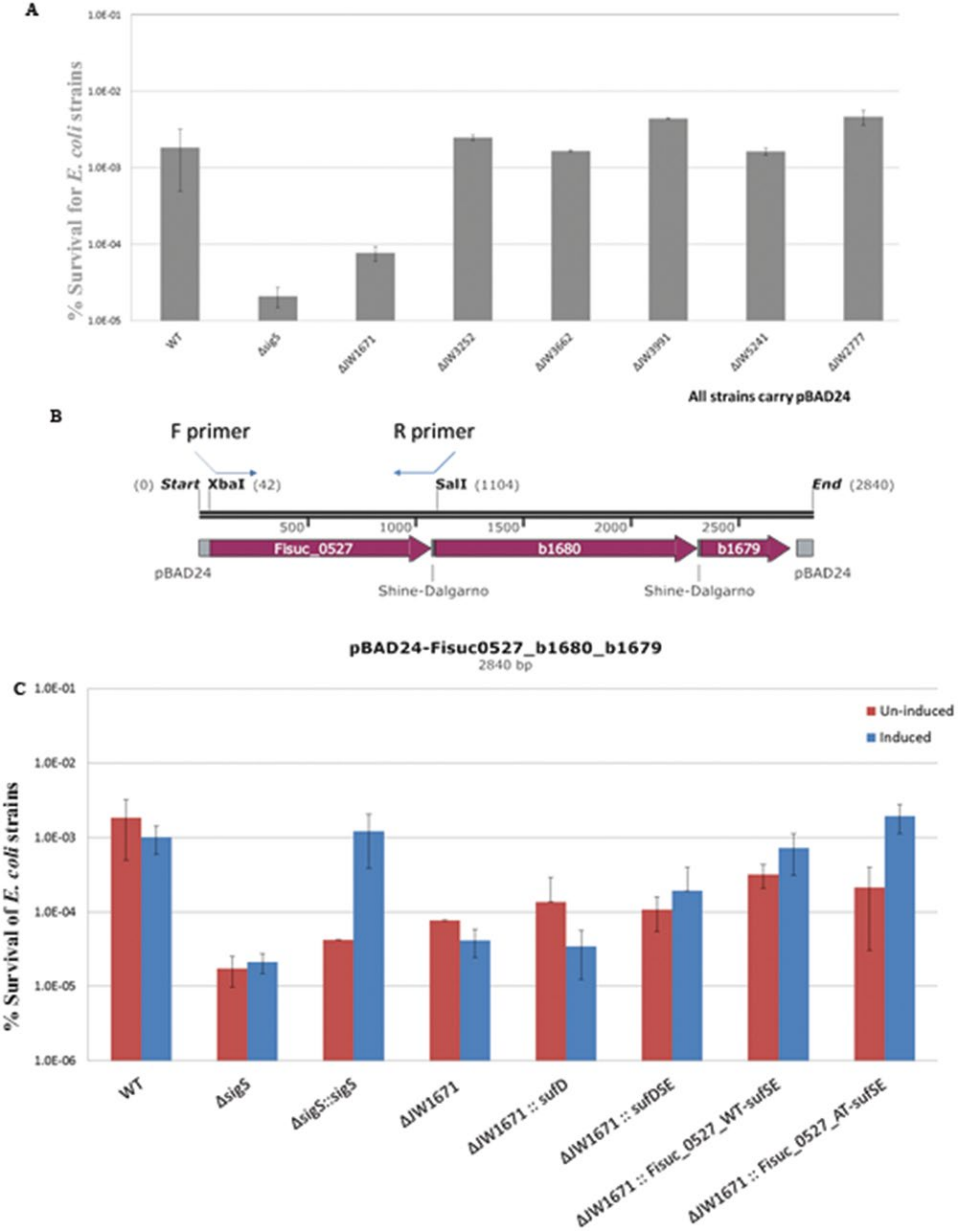
Acid survival assay of ΔJW1671 and complemented strains in *E*. *coli*. To investigate the roles of selected genes in the acid tolerant phenotype, *E*. *coli* strains deleted in individual genes homologous to acid tolerant *F*. *succinogenes* mutant genes were assayed using an acid survival CFU count method. A Δ*sigS* mutant was included as the acid sensitive control. (**A**) Among the six mutants, only the ΔJW1671 (*sufD*) mutant showed reduced ability to survival pH 2.5 treatment in minimal medium, compared to the wild type. (**B**) A linear map of the inset fragment of the fusion operon construct that was composed of *F*. *succinogenes sufD* fused with *E*. *coli sufSE*. A construct of *sufDSE* operon was first generated, and the *sufD* sequence was then replaced with the *F*. *succinogenes* counterpart. The reverse primer was designed to maintain the ribosome binding site of the immediate downstream gene. (**C**) Complementation of *E*. *coli sufDSE* but not *sufD* partially restored acid survival percentage, probably because the disruption of *sufD* caused a polar effect. Complementation with wild-type or acid tolerant *F*. *succinogenes sufD* restored the function of *E*. *coli* SufDSE, suggesting a possible role *F*. *succinogenes sufD* was involved in acid homeostasis.

To date, genetic manipulation tools are not available to construct targeted *Fibrobacter* gene knockout mutants. To investigate the function of individual genes, we used *E*. *coli* deletion strains for genes with homology to *F*. *succinogene*s genes. The six mutant genes we identified in the acid tolerant *F*. *succinogenes* all have annotated functions and homologs in wild-type *E*. *coli* BW25113 strain (Table 1). Initially acid survival percentage for each *E*. *coli* gene knockout mutant was assayed in minimal medium M9, and an acid sensitive Δ*sigS* mutant was included as a control^54^. As shown in Fig. 5(A), all the mutant strains except ΔJW1671 (Δ*sufD*) showed comparable acid survival percentages to the wild type, while the Δ*sufD* mutant survival colony count was only 2.7% of the wild type. We constructed a strain where the *E*. *coli sufD* in the *sufDSE* operon was replaced with the *F*. *succinogenes sufD* coding sequence and the coding frames and ribosome binding sites were retained (Fig. 5B). A Δ*sufD* mutant complemented with *E*. *coli sufD* alone did not restore its acid survival (Fig. 5C), likely due to polar effects on the *sufSE* genes in the *sufABCDSE* operon. The Δ*sufD* mutant complemented with the *sufDSE* three-gene operon partially restored the acid survival percentage. Although the baseline of acid survival of the Δ*sufD* mutant strain (without arabinose induction) seemed to be higher than the Δ*sigS* mutant, when induced, the complemented strains of the *sufDSE* fusion operon also partially restored acid survival percentage, and the *sufD* sequence from the acid tolerant *F*. *succinogenes* had a slightly better restoration than from the wild type.

## Discussion

We demonstrated that adapted *F*. *succinogenes* strains are capable of steady state growth in continuous culture conditions at pH 5.75 and confirmed they were able to grow in the range of pH 5.60–5.65 (Fig. 1), which is lower than that which has been previously reported^30^. It is widely thought that the lower external pH limits for continuous growth of most ruminal bacteria are between 5.9 and 6.0^29, 30, 44^. While industrial fermentative microorganisms, such as *Zymomonas mobilis* and yeast produce ethanol in the ranges pf pH 3.5–7.5 and pH 2–6.5, respectively^3^, anaerobic cellulolytic bacteria are not generally thought to grow below the pH 6.0 range^5^ and the results of this study may have broader implications for developing new biocatalysts.

Bacteria maintain their internal pH in face of changing external environments by mechanisms and responses generally called pH homeostasis. Using RNA-seq and resequencing techniques, this study has provided for the first time global insights into how *F*. *succinogenes* responds and adapts to growth in lower pH environments. When the external pH is lowered from 7.0 to 6.0, the internal pH of *F*. *succinogenes* is kept about 6.5^55^. Additionally, it is slightly lower than 6.5 when the external pH is between 5.7–6.0, whereas it drops linearly as the external pH drops to a range lower than 5.7^55^. Maintaining a pH gradient (ΔpH) across the membrane is of physiological importance because many cellular processes such as nutrient or ion transportation require the proton motive force (PMF) generated by transmembrane ΔpH and transmembrane electrical potential (Δψ)^56^. In low pH growth conditions, most bacteria take general strategies to counter the increased pH gradient across the membrane. These strategies for anaerobes include efflux of protons by proton pumps such as proton-coupled ATPases and cation-proton antiporters such as Na^+^/H^+^ and K^+^/H^+^ antiporters. In *F*. *succinogenes*, a V-type ATPase complex was identified (Fisuc_2835-Fisuc_2842), but only Fisuc_2839 (*atpD*) was significantly up-regulated during our acid treatment in the chemostat. AtpD is likely to contribute to the ATPase activity and Fisuc_2840 (*atpI*) may confer the proton transmembrane transporter activity, according to sequence analysis. However, given the observation that only one gene in the complex was up-regulated, it is not clear whether the activity of the putative proton pump was impacted during acid treatments or if this subunit was affected at the protein level. We also examined the expression of all annotated Na^+^/H^+^ and H^+^/Cl^−^ antiporters. Although not statistically significant (P > 0.50 for both pHs), only one out of five genes was up-regulated in both pH conditions, while others were down-regulated (between 1.3 and 2.5 fold). A role for antiporters for acid resistance in this case is unclear.

Another pH homeostasis strategy is to alter metabolic networks to consume or reduce production of protons. For example, some enteric bacteria up-regulate glutamate and arginine decarboxylases to consume protons, and some anaerobic bacteria such as the facultative *Streptococcus mutans* increases the activity of malolactic fermentation to incorporate protons into lactate^57^. *F*. *succinogenes* lacks glutamate decarboxylases and the malolactic fermentation pathway, and the expression levels of arginine decarboxylases, Fisuc_2975 and Fisuc_2976, were not significantly altered at pH 6.10 and 5.65. Alternatively, we identified some significantly up-regulated genes that are involved in amino acid metabolism and those pathways possibly contribute to proton consumption. A glutamate synthase NADH/NADPH subunit, Fisuc_1818, which is involved in converting a glutamine and a 2-oxoglutarate into a glutamate with participation of an NADH and a proton, was up-regulated by 3.6 fold. We also found that the two NADH-quinone oxidoreductases in *F*. *succinogenes*, Fisuc_1491 and Fisuc_2345, were up-regulated by 5.3 and 2.2 fold, respectively, only at pH 5.65. In addition, a D-cysteine disulfhydrase (Fisuc_2650) that converts a cysteine to a hydrogen sulfide, ammonia and pyruvate was up-regulated only at pH 5.65 by 2.2 fold. This reaction may compensate for reduced pyruvate production via glycolysis and generate ammonia molecules, which in turn combines with a proton. Ammonia may also be generated by the reaction catalyzed by a glutamine synthetase, Fisuc_1816 (up-regulated only at pH 5.65 by 2.5 fold), which converts a glutamine to an ammonia molecule, a glutamate and generates an ATP. It is worth noting that of all 501 significantly up-regulated genes at 5.65, only 43 were mapped in the KEGG database. There may be novel metabolic pathways that are responsible for intracellular pH homeostasis in *F*. *succinogenes* and certainly many genes respond to lower pH environments at the level of transcription. This study did not account for post-translational regulation of enzyme activity.

Resequencing analysis identified only eight sequence differences between the reference genome and our S85 wild-type strain (Table 1), indicating the reference genome sequence is an appropriate reference for our study and it is of high-quality. Genome sequencing analysis for the pH 5.65 tolerant strains identified seven single-nucleotide mutations in six genes (Table 2). Those genes were *xylE*, encoding a sugar transporter protein with unknown sugar substrate, *rlmM*, encoding a DNA alkylation repair enzyme, *dosC*, a diguanylate cyclase gene, *sufD*, an Fe-S cluster assembly complex component gene, *yidE*, a permease gene and *mscL*, encoding a membrane mechanosensitive channel protein. We initially suspected that *dosC*, *yidE* or *mscL* was responsible for the acid tolerance phenotype, since *dosC* is a possible signal transducer that may regulate several target genes^58^, *yidE* belongs to a permease family that may control transport of substances, altering PMF across the membrane^59^, and *mscL* may be important for membrane integrity in stress conditions^60^. Given the phylogenetic uniqueness and lack of genetic manipulating tools of *F*. *succinogenes*, we chose to perform a functional assay on a well-studied model organism to test the phenotype of single knockout mutants of each gene. Our acid survival CFU counting assay of the *E*. *coli* knockout mutants indicated that all single gene knockout mutants but the *sufD* (JW1671) mutant demonstrated comparable survival rates to the wild type in low pH minimal medium (Fig. 5A). In *E*. *coli*, *sufD* is located in the *sufABCDSE* operon and based on the genomic context, replacing the *sufD* coding region with a kanamycin cassette removed the ribosomal binding sites, altering the coding frames of the two downstream genes, and thus causing a polar effect on the operon. Therefore, the single gene knockout mutant of JW1671 should be functionally a *sufDSE* mutant, and a single gene complementation of *sufD* might not be sufficient for restoring the phenotype. This phenotype prediction was supported by the acid survival plating results of the *sufD* gene-complemented strains, where acid survival was not restored, and the complementation of the three genes, *sufDSE*, showed partial restoration of the acid survival phenotype. In *E*. *coli*, the model organism for studying the SUF system, the genes *sufS* and *sufE* are important for the complete functionality of the SUF system as the biogenesis of Fe-S-proteins starts with transferring the sulfur from a free cysteine molecule catalyzed by SufS, a cysteine desulphurase, which is associated to and enhanced by the helper protein, SufE^61^. The sulfur is subsequently clustered with Fe^2+^ or Fe^3+^ ions and transferred to the SufBCD complex by a still unclear mechanism. SufB and SufD share 45% sequence similarity in their C-terminal 150 residues^62^. Initially thought to function as scaffold proteins, they form a stable complex with SufC, an ATPase that provides energy to transfer Fe-S clusters to apo-receptors^52^. The ATPase activity of SufC is higher when complexed with SufB and SufD than when assayed alone^63^. In *in vivo* studies, deletion of *sufB*, *sufC* or *sufD* impairs the function of the Suf complex^64–66^. These findings suggest SufB and SufD are crucial for bacterial Fe-S homeostasis, which is in turn critical to many cellular functions as the Fe-S clusters are essential cofactors in proteins that play pivotal roles in gene regulation, central metabolism, electron transfer, and DNA repair, among others^51^. Moreover, SufD is also reported to be indispensable for the iron acquisition process in the Fe-S cluster assembly^62^. In our *E*. *coli* acid survival plating assay, when the *E*. *coli sufD* coding region in the *sufDSE* operon on the complementing plasmid was replaced with wild-type Fisuc_0527 sequence, the survival percentage was improved as compared to the Δ*sufD* mutant. This indicates *F*. *succinogenes sufD* gene confers equivalent functions in the *E*. *coli* Suf machinery. Although replacing *E*. *coli sufD* with the mutant Fisuc_0527 sequence did not further increase the survival percentage significantly, further investigation into the arginine to cysteine mutation at the 172th residue in Fisuc_0527 may be an avenue for future amino acid substitution, structural and functional studies since other mutant proteins alone did not seem to have an effect on *E*. *coli* acid survival (Fig. 5C). The mutation in Fisuc_0527 may stabilize the protein structure or also the entire SufBCD complex structure, to maintain function at lower pH. Alternatively, but not exclusively, the mutation could improve iron acquisition during the Fe-S assembly process in low pH conditions and maintain regular cellular activities. It is also worth noting that in *F*. *succinogenes*, *sufD* (Fisuc_0527), *sufS* (Fisuc_0172, Fisuc_1055) and *sufE* (Fisuc_0329) are not in one operon as seen in *E*. *coli*. Those counterparts may execute similar functions deduced by their sequence homology, but the gene regulation mechanism is expected to be distinct. The possibility that the *F*. *succinogenes* acid tolerant phenotype results from the synergistic effect of multiple mutations cannot be ruled out and combinations of multiple-gene knockout mutants in *E*. *coli* could be assayed in the future.

When monitoring metabolite concentrations in the chemostat including cellobiose, we observed a slight increase in succinate, from 11.4 mM to 12.7 mM when pH was dropped to 5.65, as well as an acetate concentration increase and formate concentration decrease, which we interpret to be the cells rebalancing the energetics and metabolism of the system. The increased yield of succinate and acetate may be of interest for industrial applications. This result, combined with our observation that batch culture at a lower pH tended to have a slight positive pressure built up in the head space, suggests that the bacteria have an altered metabolic strategy and output. This is supported by our transcriptomic analysis that the glycolysis pathway was depressed in samples from low pH chemostat, although an alternative metabolic pathway remains to be fully elucidated. We suspect that the produced gas, possibly CO_2_, partially accounts for the carbon output in the low pH chemostat culture.

In the batch culture setting with biomass as carbon source, the wild type and acid-tolerant strains produced comparable amount of succinate, regardless of the medium pH. Although the acid-tolerant population was shown to be able to survive much more acidic environments, the limiting factors for biomass utilization appear to be, instead of long-term survival, the effects of pH on bacterial attachment to substrates, biofilm formation and cellulose activities, given the proposed model that *F*. *succinogenes* requires close contact to perform cellulose deconstruction by its array of secreted cellulases^28^. In addition, analysis of the leftover biomass sugar component after fermentation in batch culture indicated that utilizations of galactose and arabinose, although only account for a minor part of the carbon energetics, were higher in pH 6.70 than in pH 5.75. It is thought that *F*. *succinogenes* has incomplete pathways to utilize galactose, mannose, fructose and pentose, and cannot survival solely on those sugars^28^; however, when incubating with glucomannan or type II arabinogalactan (which contain various linkages such as β-1,4-Man and β-1,3-Gal), *F*. *succinogenes* was found to produce 0.4 mM and 0.2 mM succinate, respectively^28^. This may explain that although they are not the major carbon sources, galactose and arabinose could be utilized to a limited extent. The utilization of those sugars, including xylan digestion, is likely also affected by pH, therefore, differences of these sugar content were observed at two different pH values. Among the *F*. *succinogenes* cellulases that have been assayed before, optimal pH for these enzymes ranged between 5.0 and 6.5^67–74^. Some of those enzymes retained 80% activities between pH 5.5 and 8.0^73, 74^. On the other hand, a fewer number of characterized xylanases showed an optimal pH at 6.0 or 6.5^75, 76^, and also retained most activities at a wide range of pH. It is thus difficult to clarify how pH affects utilization of individual sugar since the pH kinetics of most of those enzymes remain unclear, and the rest of the predicted enzymes have not been characterized.

This study has provided global insights into *F*. *succinogenes* under carbon limited continuous growth conditions at different pH levels and has shown it is possible to generate acid tolerant strains of cellulolytic anaerobic bacteria. Recent genetic engineering approaches (e.g. refs 77–79) should be adapted to enable further progress in our understanding of *F*. *succinogenes*.

## Electronic supplementary material

Supplementary Dataset Number 1
